# Factors associated with preventable infant mortality in 2020: a Brazilian population-based study

**DOI:** 10.1590/0034-7167-2023-0072

**Published:** 2024-09-20

**Authors:** Mikaelly Rayanne Moraes Sousa, Cristina Maria Garcia de Lima Parada, Hélio Rubens de Carvalho Nunes

**Affiliations:** I Universidade Estadual Paulista Júlio de Mesquita Filho. Botucatu, São Paulo, Brazil

**Keywords:** Infant Mortality, Vital Statistics, Epidemiological Studies, Health Information Systems, Health Care Quality, Mortalidad Infantil, Registros de Mortalidad, Epidemiología, Sistemas de Información en Salud, Indicadores de Calidad de Vida

## Abstract

**Objectives:**

to identify factors associated with preventable infant deaths, classified as neonatal and post-neonatal.

**Methods:**

this is an epidemiological and population-based study relating to 2020. Data from the Mortality Information System (MIS) and the preventability classification proposed in the Brazilian Health System List of Causes of Deaths Preventable by Interventions were used.

**Results:**

prematurity, living in the North and Northeast regions and the occurrence of the event at home were associated with preventable neonatal deaths. To the avoidable post-neonatal component, death outside the hospital, low maternal education and children of brown or yellow color/race were associated.

**Conclusions:**

the main risk factor associated with preventable deaths was prematurity, in the case of neonatal death. Low maternal education and occurrence outside the hospital were associated with post-neonatal deaths.

## RESUMEN

**Objetivos:**

identificar factores asociados a muertes infantiles evitables, clasificados en neonatales y posneonatales.

**Métodos:**

se trata de un estudio epidemiológico y poblacional del año 2020. Se utilizaron datos del Sistema de Información de Mortalidad (SIM) y la clasificación de evitabilidad propuesta en el Listado de Causas de Muertes Prevenibles por Intervenciones del Sistema Único de Salud.

**Resultados:**

la prematuridad, vivir en las Regiones Norte y Nordeste y la ocurrencia del evento en el domicilio se asociaron con muertes neonatales evitables. El componente posneonatal prevenible se asoció con muerte fuera del hospital, baja educación materna y niños de color/raza parda o amarilla.

**Conclusiones:**

el principal factor de riesgo asociado a muertes evitables fue la prematuridad, en el caso de la muerte neonatal. La baja educación materna y la ocurrencia fuera del hospital se asociaron con muertes posneonatales.

## INTRODUCTION

The infant mortality rate, which measures the risk of death of live births during their first year of life, reflects the social conditions of the population and their vulnerabilities related to socioeconomic conditions, quality of care and access to health^(1)^. It can be classified into neonatal components, referring to deaths of live births up to 27 days of life, and post-neonatal, which includes deaths of live births between 28 and 364 completed days^(2)^.

In 2018, the World Health Organization (WHO) defined strategic priorities for reducing child mortality: developing evidence-based and effective interventions to improve newborns’ and children’s survival and health; ensuring quality care during pregnancy, childbirth and the postnatal period; strengthening sectors such as nutrition, education, energy and social protection, which allow improvements in newborns’ and children’s health; and investing in well-coordinated policies and services^(3)^. These interventions aim to prevent potentially preventable deaths.

Deaths that could be prevented by adequate health care and guarantee of quality care in prenatal care, childbirth and the postpartum period are considered avoidable, especially as a result of effective and early diagnoses and interventions, for planning actions aimed at reduction^(4)^. Deaths with prevention potential are called sentinel events and their occurrence should trigger a detailed investigation to understand the factors that led to this outcome, since, possibly, its occurrence reflects failures in health care and indicates the need for improvement in the sectors involved in care^(5)^.

Despite all efforts, millions of children still die from preventable causes around the world^(6)^. Thus, the need to delve deeper into the issue of preventable death led authors in several countries to develop lists for classifying causes of preventable deaths as tools for preventing and detecting failures in health care^(7-13)^. Due to the great regional inequality in the distribution of maternal and child health care, using instruments that help in monitoring preventable deaths may be relevant to assess the population’s access and quality of health services in all regions^(14)^.

Thus, the most recent Brazilian classification of preventable deaths, the List of Causes of Deaths Preventable by Interventions within the scope of the Brazilian Health System (SUS - *Sistema Único de Saúde*), adopted in the present study, was proposed in 2007 and updated in 2010, enabling the investigation of neonatal and post-neonatal deaths, adopting, as preventable events, only those that can be reduced by technologies available in the SUS^(12-13)^.

There is evidence that factors associated with infant death vary depending on the age at which the event occurs^(15)^. In the neonatal period, the determinants focus mainly on quality of health care^(16)^, and in the post-neonatal period, on social and demographic determinants^(17)^. In this way, the present study may contribute to understanding the factors associated with preventability in both components - neonatal and post-neonatal-, with the potential to support the implementation of effective interventions for each of them.

## OBJECTIVES

To identify factors associated with preventable infant deaths, classified as neonatal and post-neonatal.

## METHODS

### Ethical aspects

Ethical aspect preservation was ensured, in accordance with the Brazilian National Health Council Resolution 510 of April 7, 2016, single paragraph, which states that research that uses publicly accessible information, under the terms of Law 12,527 of November 18, 2011, will not be registered or assessed by the Research Ethics Committee/Brazilian National Research Ethics Committee (CEP/CONEP - *Comitê de Ética em Pesquisa/Comissão Nacional de Ética em Pesquisa*) system, in item II^(18)^. Therefore, considering that this was research using a publicly accessible database, it was not necessary to forward it for consideration by CEP/CONEP. Therefore, the Informed Consent Form was not applicable.

### Study design

This is an epidemiological and population-based study, which adopted the STrengthening the Reporting of OBservational studies in Epidemiology (STROBE) from the EQUATOR network as a framework. Secondary data from the Mortality Information System (MIS) on infant mortality in Brazil in 2020 was used, obtained from the Brazilian Health System Information Technology Department (DATASUS - *Departamento de Informática do Sistema Único de Saúde*).

### Population and selection criteria

The study population was made up of all children who died within 364 days of life, i.e., under one year of age, totaling 31,439 cases. In data analysis and construction, variables such as codes of the establishment and municipality of occurrence, death during childbirth, fetal losses/previous miscarriages, maternal occupation, medical assistance, necropsy and attestation were excluded.

To compose the sample, a filter was applied to exclude fetuses with a gestational age of less than 22 weeks and weighing less than 1,500 g, resulting in 17,401 cases. The variables mothers’ region of residence, age and education, number of living children, type of pregnancy and birth, children’s sex and color/race, gestational age and birth weight, and location and underlying cause of death were used. Subsequently, all cases in which there was no information in the field or in which the option ignored as an answer were excluded, and only cases in which all answers were complete were used. Thus, the final sample consisted of 9,686 records and was subsequently analyzed, classifying deaths as neonatal and post-neonatal. A total of 5,127 cases of preventable infant death were analyzed.

It is noteworthy that the exploration of the full database did not reveal an association between the subset of subjects with missing data and the outcome and, therefore, despite its occurrence, the base actually used remained large, indicating that the asymptotic efficiency property of maximum likelihood estimators was valid and the estimator was unbiased.

### Study protocol

The exposure variables used in this study relate to maternal sociodemographic data, death and child characteristics, obtained from the MIS. To analyze death preventability o(outcome variable), the SUS List of Causes of Deaths Preventable by Interventions in children under 5 years of age was used, which is based on the tenth edition of the International Classification of Diseases (ICD-10), following the classifications: reducible by immunoprevention action; reducible by adequate care for women during pregnancy, childbirth, fetus and newborn; reducible by appropriate diagnostic and treatment actions; reducible by appropriate health promotion actions, linked to adequate health care actions; ill-defined causes of death; and other causes (not clearly avoidable)^(13)^.

### Analysis of results, and statistics

The investigation of factors associated with preventable death was carried out by adjusting multiple regression models with a Poisson response in two stages. First, a multiple regression model was adjusted, including, in the deterministic component, all explanatory variables. The variables that showed an association with p<0.20 were considered in the second stage, which consisted of adjusting a new multiple linear regression model with Poisson response only with the variables identified in the previous step. In the final model, associations were considered statistically significant if p<0.05. This process was replicated for each of the subpopulations formed by the combination of the year of occurrence and classification of death. Analyzes were carried out using the Statistical Package for the Social Sciences (SPSS) version 21.

## RESULTS

Table 1 relates to the characteristics of mothers, children and death for the total neonatal and post-neonatal components, without considering their classification according to preventability criteria.

**Table 1:** Characteristics of mothers, children and death for the neonatal (n=6,247) and post-neonatal (n=3,439) components according to data from the Mortality Information System, Brazil, 2023

Variables	Neonatal n (%)	Post-neonatal n (%)
Mother		
Region of residence		
North	950 (15,2)	512 (14,8)
Northeast	2.054 (32,9)	870 (25,3)
Southeast	1.930 (30,9)	1.303 (37,9)
Midwest	400 (6,4)	274 (8,0)
South	913 (14,6)	480 (14,0)
Age (years)		
≤ 19	994 (15,9)	595 (17,3)
20-34	4.061 (65,0)	2.140 (62,2)
35-40	945 (15,1)	543 (15,8)
≥ 41	247 (4,0)	161 (4,7)
Education (years)		
None	217 (3,5)	122 (3,5)
1 to 3	268 (4,3)	156 (4,5)
4 to 7	1.231 (19,7)	791 (23,1)
8 to 11 ≥ 12	3.580 (57,3) 951 (15,2)	1.840 (53,5) 530 (15,4)
Living children		
None	1.275 (20,4)	511 (14,8)
1 to 3	4.378 (70,1)	2.493 (72,5)
≥ 4	594 (9,5)	435 (12,7)
Pregnancy		
Multiple	236 (3,8)	135 (3,9)
Single	6.011 (96,2)	3.304 (96,1)
Type of birth		
Cesarean section	3.835 (61,4)	2.011 (58,5)
Vaginal	2.412 (38,6)	1.428 (41,5)
Child		
Sex		
Male	3.578 (57,3)	1.880 (54,7)
Female	2.669 (42,7)	1.559 (45,3)
Color/race		
Indigenous	117 (1,9)	94 (2,7)
Brown	3.486 (55,8)	1.598 (46,5)
Yellow	14 (0,2)	7 (0,2)
Black	139 (2,2)	129 (3,8)
White	2.491 (39,9)	1.611 (46,8)
GA at birth (weeks)		
22 to 27	32 (0,5)	10 (0,3)
28 to 31	501 (8,0)	91 (2,6)
32 to 36	2.181 (34,9)	910 (26,5)
37 to 41	3.471 (55,6)	2.380 (69,2)
≥ 42	62 (1,0)	48 (1,4)
Weight at birth (grams)		
1,500 to 2,499	2.678 (42,8)	11.260 (32,7)
2,500 to 3,999	3.322 (53,2)	2.187 (63,6)
≥4,000	247 (4,0)	126 (3,7)
Death		
Place of occurrence		
Other places	43 (0,7)	44 (1,3)
Public roads	34 (0,5)	24 (0,7)
Home	102 (1,7)	258 (7,5)
Other services	83 (1,3)	172 (5,0)
Hospital	5.985 (95,8)	2.941 (85,5)
Avoidable		
Yes	3.705 (59,3)	1.422 (41,3)
No	2.542 (40,7)	2.017 (58,7)
Preventability classification		
Reducible by immunoprevention	00 (0,0)	08 (0,2)
Reducible by adequate care during pregnancy	1.073 (17,2)	103 (3,0)
Reducible by adequate care during childbirth	1.065 (17,0)	76 (2,2)
Reducible by adequate care for the newborn	1.410 (22,6)	153 (4,5)
Reducible by diagnostic and treatment actions	42 (0,7)	537 (15,6)
Reducible by health promotion actions	115 (1,8)	545 (15,8)

*GA - gestational age.*

Regarding maternal characteristics related to the neonatal component, the Northeast region had the highest concentration of deaths (32.9%). Death was more common among women with 8-11 years of education (57.3%) and in children born by cesarean section (61.4%). Regarding children characteristics, those of brown race/color (55.8%), who were born at term (37-41 weeks) (55.6%) and weighing between 2,500 g and 3,999 g (53.2%) more frequently died. Concerning death preventability, 59.3% of neonatal deaths were considered avoidable and, of these, the majority were reducible by adequate care for newborns (26.5%), reducible by adequate care during pregnancy (17.2%), or reducible by adequate care during childbirth (17.0%) (Table 1).

In relation to maternal characteristics, in the post-neonatal component, the Southeast region had the highest concentration of deaths (37.9%). Women with 8-11 years of education were more prevalent (53.5%), and those born by cesarean section, 58.5%. In relation to children characteristics, deaths were more frequent among those of white race/color (46.8%), among full-term children (69.2%) and among those born weighing 2,500 g to 4,000 g (63.6%). As for the preventability of deaths occurring in the post-neonatal period, 41.3% of cases were considered avoidable, 15.8% were classified as reducible by health promotion actions and 15.6% as reducible by diagnostic and treatment actions (Table 1).

Table 2 refers to the unadjusted and adjusted analyzes relating to preventable neonatal deaths in 2020.

**Table 2:** Multiple regression and adjusted multiple regression relating to preventable neonatal deaths that occurred in 2020 (n=3,705) according to data from the Mortality Information System, Brazil, 2020

Variable	Multiple regression			Adjusted multiple regression		
	PR	95%CI	*p* value	PR	95%CI	*p* value
Region of residence						
North	1.31	1.15-1.49	**<0.001**	1.32	1.15-1.50	**<0.001**
Northeast	1.23	1.09-1.39	**0.001**	1.23	1.09-1.39	**0.001**
Southeast	1.10	0.98-1.23	0.117	1.09	0.97-1.22	0.145
Midwest	1.16	0.98-1.36	0.082	1.15	0.98-1.36	0.091
South	1.00					
Maternal age (years)						
≤19	1.04	0.95-1.14	0.437			
20-34	1.00					
35-40	0.97	0.88-1.07	0.602			
≥40	0.95	0.80-1.14	0.611			
Color/race						
Indigenous	1.06	0.84-1.35	0.606	1.08	0.85-1.36	0.539
Brown	1.07	0.99-1.16	0.105	1.07	0.98-1.16	0.113
Yellow	1.00	0.50-2.00	0.995	1.02	0.51-2.05	0.948
Black	1.10	0.88-1.37	0.414	1.10	0.88-1.37	0.409
White	1.00					
Education years						
None	1.08	0.89-1.32	0.432	1.09	0.90-1.33	0.368
1-3	1.10	0.92-1.31	0.311	1.11	0.93-1.33	0.234
4-7	1.10	0.98-1.24	0.112	1.12	1.00-1.26	0.049
8-11	1.06	0.95-1.17	0.287	1.07	0.97-1.18	0.185
≥ 12	1.00					
Place of death						
Other places	1.15	0.81-1.63	0.426	1.18	0.83-1.67	0.356
Public roads	1.37	0.95-1.97	0.095	1.37	0.95-1.98	0.091
Home	1.28	1.02-1.60	0.031	1.30	1.04-1.63	0.020
Other establishments	1.22	0.95-1.57	0.116	1.24	0.96-1.59	0.099
Hospital	1.00					
Number of living children						
≥ 4	1.02	0.89-1.18	0.724			
1-3	0.97	0.89-1.06	0.490			
0	1.00					
Type of pregnancy						
Doble or triple	1.22	1.04-1.43	0.017	1.19	1.01-1.39	0.036
Single	1.00					
Weeks of pregnancy						
22-27	1.43	0.96-2.13	0.077	1.34	0.90-1.98	0.151
28-31	1.49	1.31-1.69	**<0.001**	1.33	1.19-1.49	**<0.001**
32-36	1.16	1.07-1.26	**<0.001**	1.08	1.00-1.16	0.043
37-41	1.00					
≥ 42	1.29	0.97-1.71	0.085	1.31	0.99-1.75	0.060
Type of birth						
Cesarean section	0.83	0.78-0.89	**<0.001**	0.83	0.78-0.89	**<0.001**
Vaginal	1.00					
Weight at birth (grams)						
1,500-2,499	0.86	0.80-0.94	**0.001**			
2,500-3,999	1.00					
≥ 4,000	1.07	0.91-1.27	0.400			
Children’s sex						
Male	1.05	0.99-1.12	0.128	1.06	0.99-1.13	0.098
Female	1.00					

*PR - Prevalence Ratio; 95%CI - 95% Confidence Interval.*

It is observed, in Table 2, that the variable birth weight presented p<0.20, but was not included in the final model, as it presented collinearity with the variable gestational age at birth. Independently, being born in the North (PR: 1.32; 95%CI: 1.15-1.50; p: <0.001) or Northeast (PR: 1.23; 95%CI: 1.09-1.39; p: 0.001), being born at home (PR: 1.30; 95%CI: 1.04-1.63; p: 0.020), being a mother with 4-7 years of education (PR: 1.12; 95%CI: 1.00 -1.26; p: 0.049), multiple pregnancy (PR: 1.19; 95%CI: 1.01-1.39; p: 0.036) and being born at 28-31 weeks (PR: 1.31; 95%CI: 1.19-1.49; p: <0.001) or 32-36 weeks (PR: 1.08; 95%CI: 1.00 -1.16; p: 0.043) constituted risk factors for preventable death in the neonatal period compared to children born at term. It was a protective factor for preventable death to be born by cesarean section (PR: 0.83; 95%CI: 0.78-0.89; p: <0.001) compared to being born vaginally.

Regarding the magnitude of the effects, in the risk of preventable neonatal death, being born in the North or Northeast regions increased by 32% and 23%, respectively, compared to the South region; 4-7 years of education increased by 12% compared to 12 or more years of education; regarding the occurrence at home, the risk was 30% higher when compared to the occurrence in the hospital; Regarding multiple pregnancies, the risk was 19% higher when compared to single pregnancies; and in relation to being born prematurely, at 28-31 weeks or 32-36 weeks, the risk was 33% and 8% higher, respectively, when compared to being born at term (Table 2).

Table 3 refers to the unadjusted and adjusted analyzes relating to preventable post-neonatal death in 2020.

**Table 3:** Multiple regression and adjusted multiple regression relating to preventable post-neonatal deaths (n=1,422) that occurred in 2020 according to data from the Mortality Information System, Brazil, 2020

Variable	Multiple regression			Adjusted multiple regression		
	PR	95%CI	*p* value	PR	95%CI	*p* value
Region						
North	0.78	0.63-0.97	0.022	0.78	0.63-0.96	0.021
Northeast	0.92	0.77-1.11	0.392	0.92	0.77-1.11	0.383
Southeast	0.98	0.83-1.15	0.782	0.97	0.82-1.15	0.737
Midwest	0.83	0.65-1.06	0.137	0.82	0.64-1.05	0.124
South	1.00					
Maternal age (years)						
≤19	1.01	0.88-1.17	0.866	1.02	0.89-1.17	0.781
20-34	1.00					
35-40	0.88	0.75-1.03	0.111	0.87	0.74-1.02	0.095
>40	0.73	0.54-0.99	0.043	0.71	0.53-0.96	0.028
Color/race						
Indigenous	1.32	0.97-1.81	0.079	1.35	0.99-1.85	0.058
Brown	1.14	1.01-1.29	0.032	1.14	1.01-1.28	0.040
Yellow	2.42	1.08-5.44	0.032	2.41	1.07-5.40	0.033
Black	1.27	0.99-1.64	0.062	1.27	0.98-1.63	0.069
White	1.00					
Education years						
None	1.39	1.01-1.91	0.043	1.41	1.03-1.94	0.032
1-3	1.35	1.00-1.82	0.049	1.38	1.03-1.85	0.031
4-7	1.45	1.18-1.78	0.001	1.45	1.18-1.78	**<0.001**
8-11	1.30	1.08-1.57	0.006	1.30	1.08-1.57	0.005
≥ 12	1.00					
Place of death						
Other places	1.47	0.99-2.18	0.054	1.49	1.01-2.20	0.046
Public roads	1.69	1.03-2.79	0.039	1.68	1.02-2.77	0.042
Home	1.57	1.33-1.86	**<0.001**	1.57	1.33-1.86	**<0.001**
Other establishments	1.69	1.39-2.05	**<0.001**	1.69	1.40-2.05	**<0.001**
Hospital	1.00					
Number of living children						
≥ 4	0.97	0.79-1.20	0.809			
1-3	0.91	0.78-1.06	0.214			
0	1.00					
Type of pregnancy						
Double or triple	1.30	1.01-1.68	0.042	1.28	0.99-1.64	0.056
Single	1.00					
Weeks of pregnancy						
22-27	1.12	0.42-3.03	0.819	1.04	0.39-2.79	0.937
28-31	1.60	1.19-2.16	0.002	1.42	1.08-1.88	0.013
32-36	1.11	0.96-1.27	0.161	1.02	0.90-1.16	0.716
37-41	1.00					
≥ 42	1.22	0.83-1.79	0.305	1.27	0.87-1.85	0.220
Type of birth						
Cesarean section	0.84	0.76-0.94	0.003	0.84	0.75-0.94	0.002
Vaginal	1.00					
Birth at weight (grams)						
1,500-2,499	0.86	0.75-0.98	0.029			
2,500-3,999	1.00					
≥ 4,000	1.14	0.87-1.49	0.332			
Children’s sex						
Male	1.06	0.95-1.18	0.282			
Female	1.00					

* PR - Prevalence Ratio;

** 95%CI - 95% Confidence Interval.

According to Table 3, the birth weight variable presented p<0.20, but was not included in the final model, as it presented collinearity with the gestational age variable at birth. In the final model, independently, children were brown (PR: 1.14; 95%CI: 1.01-1.28; p: 0.040) or yellow (PR: 2.41; 95%CI: 1.07-5.40; p: 0.033) race/color, mother with no education (PR: 1.41; 95%CI: 1.03-1.94; p: 0.032) or has 1-3 years (PR: 1.38; 95%CI: 1.03-1.85; p: 0.031), 4-7 years (PR: 1.45; 95%CI: 1.18-1.78; p: <0.001) or 8-11 years (PR: 1.30; 95 %CI: 1.08-1.57; p: 0.005) of school approval, death occurred in other places (PR: 1.49; 95%CI: 1.01-2.20; p: 0.046), on public roads (PR: 1.68; 95%CI: 1.02-2.77; p: 0.042), at home (PR: 1.57; 95%CI: 1.33-1.86; p: <0.001) or other health establishments (PR: 1.69; 95%CI: 1.40-2.05; p: <0.001) and children were born with a gestational age between 28 and 31 weeks (PR: 1.42; 95%CI: 1.08-1.88; p: 0.013) constituted a risk factor for preventable death in the post-neonatal period. Being born in the North region (PR: 0.78; 95%CI: 0.63-0.96; p: 0.021), maternal age over 40 years old (PR: 0.71; 95%CI: 0.53-0 .96; p: 0.028) and born by cesarean section (PR: 0.84; 95%CI: 0.75-0.94; p: 0.002) constituted a protective factor for preventable post-neonatal death.

As for the magnitude of the effects on preventable post-neonatal death, having brown skin color increased the risk by 14% and yellow skin color by 41%; being illiterate, having 1-3, 4-7 or 8-11 years of education increased the risk by 41%, 38%, 45% and 30%, respectively; occurrence of death in other health services increased the risk by 69%, on public roads by 68%, at home by 57% and in other places by 49% when compared to death in hospital; being born at a gestational age between 28-31 weeks resulted in a 42% higher risk than being born at term. As for protective factors, children born in the North region had a 22% lower risk compared to those born in the South region; children whose mothers were 40 years old or older had a 29% lower risk compared to women aged 20-34; and being born by cesarean section reduced the risk by 16% compared to birth via vaginal birth (Table 3).

## DISCUSSION

The present study made it possible to identify factors independently associated with preventable infant deaths in 2020, considering their neonatal and post-neonatal components. For preventable neonatal deaths, being born by cesarean section was a protective factor. Gestational age of 28-36 weeks, living in the North or Northeast regions, education of 4-7 years, death at home and multiple pregnancies were risk factors. For the avoidable post-neonatal component, being born by cesarean section was a protective factor, as was being born in the North region and maternal age over 40 years. Risk factors were related to lower education levels, yellow or brown race/color, death outside the hospital and being born at 28-31 weeks of gestation.

In the present investigation, living in the North and Northeast regions was a risk factor for neonatal death, in agreement with other studies, which showed that the North and Northeast regions have the worst neonatal mortality rates in the country^(19-20)^. Furthermore, it is noteworthy that, in northeastern Brazil, many states present stationary behavior in relation to infant mortality, a worrying fact, since the mortality rate is still high^(21)^ and that, in Rondônia, in the North region, the most relevant reduction in the country was found when considering preventable neonatal deaths from 2000 to 2018^(22)^.

Being born prematurely, at 28-36 weeks, was a risk factor for preventable neonatal death. When addressing this component, prematurity is among the dominant causes, being considered difficult to prevent and dependent on several factors, being mainly linked to the quality of prenatal care, the organization of neonatal services and the preparation of the team from Primary Care to Tertiary Health Care so that its reduction requires investments to strengthen the health system^(23)^. Prenatal care, when carried out appropriately, is capable of reducing maternal and child morbidity and mortality, with evidence that complete prenatal care and postnatal care are related to a reduction in early neonatal mortality^(24)^.

Prematurity as a cause of infant death is relevant in the global context, as, in 2022, it contributed to 35% of global neonatal mortality^(25)^, with its complications being the main causes of death in all regions of the world^(26)^, including in developed countries. In Serbia, the neonatal mortality rate related to prematurity increased from 7.2% in 2000 to 11.9% in 2014^(27)^. Among its complications, respiratory distress syndrome and birth asphyxia stand out, as fetal lung development is interrupted in prematurity^(28)^. Therefore, it is not surprising that these are the main causes of neonatal death in the world, especially because, in addition to cases of prematurity that occur naturally, there are also medical conditions in which it is necessary to shorten the pregnancy for maternal and/or fetal reasons. Therefore, despite being complex, given its multifactorial nature^(29)^, it is essential to prevent prematurity in order to reduce avoidable neonatal deaths.

It should also be noted that the present study used a classification of preventability of infant death, which only includes cases of children born weighing at least 1,500 g, which certainly excluded many cases of premature births, not considered here subject to classification according to the adopted preventability criteria. On the other hand, it is also worth highlighting that a gestational age of 28-36 weeks was also a risk factor for post-neonatal death, and this period possibly allowed the inclusion of premature infants weighing 1,500 g or more.

Multiple pregnancies were associated with preventable neonatal death, a result in agreement with that obtained in a Korean study, with twin pregnancies increasing the risk of neonatal death by nine times and the risk of infant death by six times in general when compared to a single pregnancy, and the authors pointed out that the risk increases exponentially in the case of triplets or quadruplets^(30)^.

Furthermore, intermediate education and 4-7 years of school approval deserve to be explored in future studies, as they were risk factors for preventable neonatal death, a result that is difficult to explain, and deaths occurring at home, because, in addition to the difficulty of timely access to quality health services^(31-32)^, other aspects need to be considered in the context of preventability, such as length of pregnancy (prematurity).

Globally, there was a 50% reduction in overall post-neonatal deaths between 1990 and 2015^(15)^. Post-neonatal death tends to have causes that are more easily amenable to intervention, as it is related to living conditions and family characteristics, such as socioeconomic conditions, education, basic sanitation and treated water, in addition to increasing health programs, vaccination coverage and combating communicable infectious diseases^(33-34)^. In this regard, the associated factors found in this study that are related to social aspects are education and death outside the hospital, due to their relationship with access to health services.

Having brown or yellow skin color/race was a risk factor for post-neonatal death, and when the color was yellow, there was twice the risk of death, a fact that needs to be viewed with caution, due to the small number of cases (n=7) and because studies show a lower mortality rate in this population^(35-36)^. In the United States of America, black children die three times more often than white children^(37)^, a relationship not observed in the present study. In a Brazilian study, carried out in the state of Pernambuco, it was observed that black and brown children were 13 times more associated with neonatal death^(38)^ and, in Mato Grosso do Sul, brown children showed an increase in neonatal deaths between 2005 and 2013^(39)^.

It was found that being born by cesarean section was a protective factor for preventable death, both when considering the neonatal and post-neonatal components. This result is in agreement with that obtained by a study that focused on comparing the modes of birth and found that, in general, the majority of neonatal deaths occurred after vaginal birth, and may be associated with care practices^(40)^. It is possible that using harmful procedures and interventions during childbirth may increase children’s risk of death, but unindicated cesarean sections may also terminate the pregnancy beforehand, consequently increasing the number of premature births^(41)^. Studies have identified the same protective factor as cesarean section on infant mortality in general, that is, without considering the preventability criteria^(42-43)^. However, it was not possible, in this study, to identify whether the cesarean section was elective or emergency, an essential condition for understanding exactly the protective role of the cesarean section.

For the post-neonatal component, living in the North region protected against avoidable death, a situation that deserves to be better investigated in future studies. Maternal age over 40 years also protected the occurrence of preventable deaths, i.e., the majority of deaths that occurred in this age group were not due to preventable causes. This may occur because advanced maternal age is associated with a greater risk of congenital and chromosomal abnormalities, which result in fetal complications^(44)^. It is noteworthy that, in most cases, the association between malformation and infant death is difficult to prevent, and the indication of screening and early interruption is controversial, which is often the only option^(45)^. Unlike the present study, in a Korean study, it was observed that maternal age over 40 years was associated with a higher risk of infant death, a comparison that needs to be made with care, since, in the case of Korea, death was addressed in general and not according to preventability criteria^(46)^.

### Study limitations

A weakness of this study is the fact that it used a secondary database, which does not allow data collection control. However, despite the need to exclude part of the cases, the base actually used still remained large, possibly indicating an unbiased estimator. The fact that a population-based database from a country like Brazil, which has continental dimensions, was used is considered a power to be highlighted. Another weakness to be highlighted is the fact that we worked with the proportion of preventable deaths, not calculating mortality rates, which are an indicator frequently used in studies on this topic.

### Contributions to nursing

Studies on death preventability are relevant to nursing, as nurses have an essential role in investigating infant deaths and classifying death preventability in mortality committees. Thus, the results obtained signal the power of the nursing area in proposing and implementing public health policies that encompass adequate and quality access, from pregnancy to early childhood, expanding the view of this population and articulating with the technologies available in the health system, with the aim of contributing to reducing preventable infant mortality in Brazil.

## CONCLUSIONS

The results made it possible to identify the determinants that were associated with neonatal and post-neonatal deaths. For preventable neonatal deaths, being born by cesarean section was an independent protective factor, whereas prematurity was a risk factor. Living in the North and Northeast regions, having low education and occurrence of death at home also constituted independent risk factors for the negative outcome. In the post-neonatal period, maternal education, deaths occurring outside the hospital and brown and yellow race/color constituted risk factors for preventable death. Being born by cesarean section was an independent protective factor as well as living in the North region and maternal age over 40 years old.

Considering the magnitude of the effects, prematurity stood out for preventable neonatal death. For preventable post-neonatal death, the social risk stood out, represented by low maternal education and difficulty in accessing health services, evidenced by the occurrence of death outside hospital settings.
