# Supplementary material for: Factors associated with preventable infant mortality in 2020: a Brazilian population-based study
Source: Rev Bras Enferm. 2024 Sep 20;77(4):e20230072. doi: 10.1590/0034-7167-2023-0072 (PMC11419689; doi:10.1590/0034-7167-2023-0072)
Supplement: Translation

# Translation: Fatores associados à mortalidade infantil evitável no ano de 2020: estudo brasileiro de base populacional

**DOI:** 10.1590/0034-7167-2023-0072pt

**Authors:** Mikaelly Rayanne Moraes Sousa, Cristina Maria Garcia de Lima Parada, Hélio Rubens de Carvalho Nunes

**Affiliations:** I Universidade Estadual Paulista Júlio de Mesquita Filho. Botucatu, São Paulo, Brasil

**Keywords:** Mortalidade Infantil, Registros de Mortalidade, Epidemiologia, Sistemas de Informação em Saúde, Qualidade da Assistência à, Saúde

## Abstract

**Objetivos:**

identificar os fatores associados aos óbitos infantis evitáveis, classificados em neonatais e pós-neonatais.

**Métodos:**

trata-se de estudo epidemiológico e de base populacional relativo ao ano de 2020. Foram utilizados dados do Sistema de Informações de Mortalidade (SIM) e da classificação de evitabilidade proposta na Lista de Causas de Mortes Evitáveis por Intervenções do Sistema Único de Saúde.

**Resultados:**

associaram-se aos óbitos neonatais evitáveis prematuridade, residir nas Regiões Norte e Nordeste e a ocorrência do evento em domicílio. Ao componente pós-neonatal evitável, associaram-se óbito fora do hospital, baixa escolaridade materna e crianças de cor/raça parda ou amarela.

**Conclusões:**

o principal fator de risco associado aos óbitos evitáveis foi a prematuridade, no caso do óbito neonatal. A baixa escolaridade materna e a ocorrência fora do hospital associaram-se aos óbitos pós-neonatais.

## INTRODUÇÃO

A taxa de mortalidade infantil, que mensura o risco de morte dos nascidos vivos durante o seu primeiro ano de vida, reflete as condições sociais da população e suas vulnerabilidades relacionadas às condições socioeconômicas, da qualidade da assistência e de acesso à saúde^(1)^. Pode ser classificada nos componentes neonatal, referente aos óbitos de nascidos vivos até 27 dias de vida, e pós-neonatal, que inclui óbitos de nascidos vivos entre 28 e 364 dias completos^(2)^.

Em 2018, a Organização Mundial da Saúde (OMS) definiu prioridades estratégicas para redução da mortalidade infantil: desenvolver intervenções baseadas em evidências e eficazes para melhorar a sobrevivência e saúde de recém-nascidos e crianças; garantir atendimento de qualidade durante a gravidez, o parto e o período pós-natal; fortalecer setores como nutrição, educação, energia e proteção social, que permitem melhorias na saúde do recém-nascido e da criança; e investir em políticas e serviços bem coordenados^(3)^. Essas intervenções visam coibir óbitos potencialmente evitáveis.

Conceituam-se como evitáveis os óbitos que poderiam ser prevenidos pela adequada atenção à saúde e garantia de assistência de qualidade no pré-natal, parto e puerpério, especialmente em decorrência de diagnósticos e intervenções efetivas e precoces, para o planejamento de ações que visam sua redução^(4)^. Óbitos com potencial de prevenção são chamados eventos sentinela e sua ocorrência deve dar início a uma detalhada investigação para compreender os fatores que levaram a esse desfecho, já que, possivelmente, sua ocorrência reflete falhas na atenção à saúde e indicam a necessidade de aprimoramento nos setores envolvidos no atendimento^(5)^.

Apesar de todos os esforços, milhões de crianças ainda morrem por causas evitáveis no mundo^(6)^. Assim, a necessidade de aprofundamento na temática de evitabilidade do óbito levou autores em diversos países a desenvolverem listas para classificação de causas de mortes evitáveis como ferramentas na prevenção e detecção de falhas na assistência à saúde^(7-13)^. Devido à grande desigualdade regional na distribuição da assistência à saúde materno-infantil, utilizar instrumentos que auxiliem no monitoramento de mortes evitáveis pode ser pertinente para avaliar o acesso da população e a qualidade dos serviços de saúde em todas as regiões^(14)^.

Nesse sentido, a mais recente classificação brasileira de óbitos evitáveis, a Lista de Causas de Mortes Evitáveis por Intervenções no âmbito do Sistema Único de Saúde (SUS), adotada no presente estudo, foi proposta em 2007 e atualizada em 2010, possibilitando a investigação de óbitos neonatais e pós-neonatais, adotando, como eventos evitáveis, apenas aqueles reduzíveis por tecnologias disponíveis no SUS^(12-13)^.

Há evidências de que os fatores associados ao óbito infantil variam, dependendo da idade em que o evento ocorre^(15)^. No período neonatal, os determinantes se concentram, principalmente, na qualidade da assistência à saúde^(16)^, e no período pós-neonatal, aos determinantes sociais e demográficos^(17)^. Dessa forma, o presente estudo poderá contribuir para a compreensão dos fatores associados à evitabilidade nos dois componentes - neonatal e pós-neonatal-, com potencial de subsidiar a implementação de intervenções efetivas a cada um deles.

## OBJETIVOS

Identificar os fatores associados aos óbitos infantis evitáveis, classificados em neonatais e pós-neonatais.

## MÉTODOS

### Aspectos éticos

Foi assegurada a preservação dos aspectos éticos, de acordo com a Resolução do Conselho Nacional de Saúde n^o^ 510, de 7 de abril de 2016, parágrafo único, o qual dispõe que não serão registradas nem avaliadas pelo sistema Comitê de Ética em Pesquisa/Comissão Nacional de Ética em Pesquisa (CEP/CONEP), no item II, pesquisas que utilizam informações de acesso público, nos termos da Lei n^o^ 12.527, de 18 de novembro de 2011^(18)^. Assim, considerando tratar-se de pesquisa com banco de dados de acesso público, não foi necessário encaminhamento para apreciação pelo CEP/CONEP. Dessa forma, o Termo de Consentimento Livre e Esclarecido não foi aplicável.

### Desenho do estudo

Trata-se de estudo epidemiológico e de base populacional, que adotou o *STrengthening the Reporting of OBservational studies in Epidemiology* (STROBE) da rede EQUATOR como referência. Utilizaram-se dados secundários do Sistema de Informações de Mortalidade (SIM) sobre a mortalidade infantil no Brasil, no ano de 2020, obtido a partir do Departamento de Informática do Sistema Único de Saúde (DATASUS).

### População e critérios de seleção

A população do estudo foi composta por todas as crianças que evoluíram para óbito até 364 dias de vida, ou seja, menores de um ano, somando 31.439 casos. Na análise e construção de dados, foram excluídas variáveis como códigos do estabelecimento e do município de ocorrência, óbito no parto, perdas fetais/abortos anteriores, ocupação materna, assistência médica, necropsia e atestante.

Para composição da amostra, foi aplicado filtro para exclusão dos fetos com idade gestacional inferior a 22 semanas e peso inferior a 1.500 g, resultando em 17.401 casos. Foram utilizadas as variáveis região de residência, idade e escolaridade da mãe, número de filhos vivos, tipo de gravidez e de parto, sexo e cor/raça da criança, idade gestacional e peso ao nascer, e local e causa básica do óbito. Em seguida, foram excluídos todos os casos em que no campo não constavam informação ou que constava a opção ignorado como resposta, sendo utilizados apenas os casos em que todas as respostas estavam completas. Assim, a amostra final foi constituída por 9.686 registros e posteriormente analisada, classificando-se os óbitos em neonatal e pós-neonatal. Foram analisados 5.127 casos de óbito infantil evitável.

Destaca-se que a exploração do banco integral não revelou associação entre o subconjunto de sujeitos com *missing* e o desfecho e, assim, apesar de sua ocorrência, a base efetivamente utilizada manteve-se grande, indicando que a propriedade da eficiência assintótica dos estimadores de máxima verossimilhança era válida, e o estimador, não viciado.

### Protocolo do estudo

As variáveis de exposição utilizadas neste estudo são relativas a dados sociodemográficos maternos, às características do óbito e da criança, obtidas do SIM. Para a análise da evitabilidade dos óbitos (variável desfecho), foi empregada a Lista de Causas de Mortes Evitáveis por Intervenções do SUS em menores de 5 anos, que é baseada na décima edição da Classificação Internacional de Doenças (CID-10), seguindo as classificações: reduzíveis por ação de imunoprevenção; reduzíveis por adequada atenção à mulher na gestação, parto, feto e recém-nascido; reduzíveis por ações adequadas de diagnóstico e tratamento; reduzíveis por ações adequadas de promoção à saúde, vinculadas a ações adequadas de atenção à saúde; causas de morte mal definidas; e demais causas (não claramente evitáveis)^(13)^.

### Análise dos resultados e estatística

A investigação dos fatores associados ao óbito evitável foi realizada ajustando modelos de regressão múltipla com reposta Poisson em duas etapas. Na primeira, foi ajustado um modelo de regressão múltipla, incluindo, no componente determinístico, todas as variáveis explicativas. As variáveis que apresentaram associação com p<0,20 foram consideradas na segunda etapa, que consistiu no ajuste de um novo modelo de regressão linear múltipla com resposta Poisson apenas com as variáveis identificadas na etapa anterior. No modelo final, as associações foram consideradas estatisticamente significativas se p<0,05. Esse processo foi replicado para cada uma das subpopulações formadas pela combinação entre o ano de ocorrência e a classificação do óbito. As análises foram feitas com o *software Statistical Package for the Social Sciences* (SPSS), versão 21.

## RESULTADOS

A Tabela 1 é relativa às características das mães, das crianças e do óbito para os componentes neonatal e pós-neonatal totais, ainda sem considerar sua classificação segundo critérios de evitabilidade.

**Tabela 1:** Características da mãe, da criança e do óbito para os componentes neonatal (n=6.247) e pós-neonatal (n=3.439) segundo dados do Sistema de Informação de Mortalidade, Brasil, 2023

Variáveis	Neonatal n (%)	Pós-neonatal n (%)
Mãe		
Região de residência		
Norte	950 (15,2)	512 (14,8)
Nordeste	2.054 (32,9)	870 (25,3)
Sudeste	1.930 (30,9)	1.303 (37,9)
Centro-Oeste	400 (6,4)	274 (8,0)
Sul	913 (14,6)	480 (14,0)
Idade (anos)		
≤ 19	994 (15,9)	595 (17,3)
20-34	4.061 (65,0)	2.140 (62,2)
35-40	945 (15,1)	543 (15,8)
≥ 41	247 (4,0)	161 (4,7)
Escolaridade (anos)		
Nenhuma	217 (3,5)	122 (3,5)
1 a 3	268 (4,3)	156 (4,5)
4 a 7	1.231 (19,7)	791 (23,1)
8 a 11 ≥ 12	3.580 (57,3) 951 (15,2)	1.840 (53,5) 530 (15,4)
Filhos vivos		
Nenhum	1.275 (20,4)	511 (14,8)
1 a 3	4.378 (70,1)	2.493 (72,5)
≥ 4	594 (9,5)	435 (12,7)
Gravidez		
Múltipla	236 (3,8)	135 (3,9)
Única	6.011 (96,2)	3.304 (96,1)
Tipo de parto		
Cesárea	3.835 (61,4)	2.011 (58,5)
Vaginal	2.412 (38,6)	1.428 (41,5)
Criança		
Sexo		
Masculino	3.578 (57,3)	1.880 (54,7)
Feminino	2.669 (42,7)	1.559 (45,3)
Cor/raça		
Indígena	117 (1,9)	94 (2,7)
Parda	3.486 (55,8)	1.598 (46,5)
Amarela	14 (0,2)	7 (0,2)
Preta	139 (2,2)	129 (3,8)
Branca	2.491 (39,9)	1.611 (46,8)
IG ao nascer (semanas)		
22 a 27	32 (0,5)	10 (0,3)
28 a 31	501 (8,0)	91 (2,6)
32 a 36	2.181 (34,9)	910 (26,5)
37 a 41	3.471 (55,6)	2.380 (69,2)
≥ 42	62 (1,0)	48 (1,4)
Peso ao nascer (gramas)		
1.500 a 2.499	2.678 (42,8)	11.260 (32,7)
2.500 a 3.999	3.322 (53,2)	2.187 (63,6)
≥4.000	247 (4,0)	126 (3,7)
Óbito		
Local de ocorrência		
Outros locais	43 (0,7)	44 (1,3)
Via pública	34 (0,5)	24 (0,7)
Domicílio	102 (1,7)	258 (7,5)
Outros serviços	83 (1,3)	172 (5,0)
Hospital	5.985 (95,8)	2.941 (85,5)
Evitável		
Sim	3.705 (59,3)	1.422 (41,3)
Não	2.542 (40,7)	2.017 (58,7)
Classificação de evitabilidade		
Reduzível por imunoprevenção	00 (0,0)	08 (0,2)
Reduzível por adequada atenção à gestação	1.073 (17,2)	103 (3,0)
Reduzível por adequada atenção ao parto	1.065 (17,0)	76 (2,2)
Reduzível por adequada atenção ao recém-nascido	1.410 (22,6)	153 (4,5)
Reduzível por ações de diagnóstico e tratamento	42 (0,7)	537 (15,6)
Reduzível por ações de promoção à saúde	115 (1,8)	545 (15,8)

*IG - idade gestacional.*

Quanto às características maternas relativas ao componente neonatal, a Região Nordeste obteve a maior concentração de óbitos (32,9%). O óbito foi mais frequente entre mulheres com 8-11 anos de estudo (57,3%) e em crianças nascidas por cesariana (61,4%). No tocante às características das crianças, tiveram mais frequente evolução para óbito aquelas de raça/cor parda (55,8%), que nasceram a termo (37-41 semanas) (55,6%) e com peso entre 2.500 g e 3.999 g (53,2%). Referente à evitabilidade do óbito, foram considerados evitáveis 59,3% dos óbitos neonatais e, desses, a maioria era reduzível por adequada atenção ao recém-nascido (26,5%), reduzível por adequada atenção à gestação (17,2%), ou reduzível por adequada atenção ao parto (17,0%) (Tabela 1).

Em relação às características maternas, no componente pós-neonatal, a Região Sudeste obteve a maior concentração de óbitos (37,9%). Mulheres com 8-11 anos de estudo foram mais prevalentes (53,5%), e os nascidos por cesariana, 58,5%. Sobre as características das crianças, os óbitos foram mais frequentes naquelas de raça/cor branca (46,8%), entre crianças a termo (69,2%) e de nascidos com peso de 2.500 g a 4.000 g (63,6%). Em relação à evitabilidade do óbito ocorrido no período pós-neonatal, foram considerados evitáveis 41,3% dos casos, sendo que 15,8% foram classificados como reduzíveis por ações de promoção à saúde e, 15,6%, reduzíveis por ações de diagnóstico e tratamento (Tabela 1).

A Tabela 2 refere-se às análises bruta e ajustada relativas ao óbito neonatal evitável no ano de 2020.

**Tabela 2:** Regressão múltipla e regressão múltipla ajustada relativas aos óbitos neonatais evitáveis ocorridos em 2020 (n=3.705) segundo dados do Sistema de Informação de Mortalidade, Brasil, 2020

Variável	Regressão múltipla			Regressão múltipla ajustada		
	RP	IC95%	Valor de *p*	RP	IC95%	Valor de *p*
Região						
Norte	1,31	1,15-1,49	**<0,001**	1,32	1,15-1,50	**<0,001**
Nordeste	1,23	1,09-1,39	**0,001**	1,23	1,09-1,39	**0,001**
Sudeste	1,10	0,98-1,23	0,117	1,09	0,97-1,22	0,145
Centro-Oeste	1,16	0,98-1,36	0,082	1,15	0,98-1,36	0,091
Sul	1,00					
Idade materna (anos)						
≤19	1,04	0,95-1,14	0,437			
20-34	1,00					
35-40	0,97	0,88-1,07	0,602			
≥40	0,95	0,80-1,14	0,611			
Cor/raça						
Indígena	1,06	0,84-1,35	0,606	1,08	0,85-1,36	0,539
Parda	1,07	0,99-1,16	0,105	1,07	0,98-1,16	0,113
Amarela	1,00	0,50-2,00	0,995	1,02	0,51-2,05	0,948
Preta	1,10	0,88-1,37	0,414	1,10	0,88-1,37	0,409
Branca	1,00					
Anos de escolaridade						
Nenhum	1,08	0,89-1,32	0,432	1,09	0,90-1,33	0,368
1-3	1,10	0,92-1,31	0,311	1,11	0,93-1,33	0,234
4-7	1,10	0,98-1,24	0,112	1,12	1,00-1,26	0,049
8-11	1,06	0,95-1,17	0,287	1,07	0,97-1,18	0,185
≥ 12	1,00					
Local do óbito						
Outros	1,15	0,81-1,63	0,426	1,18	0,83-1,67	0,356
Via pública	1,37	0,95-1,97	0,095	1,37	0,95-1,98	0,091
Domicílio	1,28	1,02-1,60	0,031	1,30	1,04-1,63	0,020
Outros estabelecimentos	1,22	0,95-1,57	0,116	1,24	0,96-1,59	0,099
Hospital	1,00					
Número de filhos vivos						
≥ 4	1,02	0,89-1,18	0,724			
1-3	0,97	0,89-1,06	0,490			
0	1,00					
Tipo de gravidez						
Dupla ou tripla	1,22	1,04-1,43	0,017	1,19	1,01-1,39	0,036
Única	1,00					
Semanas de gestação						
22-27	1,43	0,96-2,13	0,077	1,34	0,90-1,98	0,151
28-31	1,49	1,31-1,69	**<0,001**	1,33	1,19-1,49	**<0,001**
32-36	1,16	1,07-1,26	**<0,001**	1,08	1,00-1,16	0,043
37-41	1,00					
≥ 42	1,29	0,97-1,71	0,085	1,31	0,99-1,75	0,060
Tipo de parto						
Cesárea	0,83	0,78-0,89	**<0,001**	0,83	0,78-0,89	**<0,001**
Vaginal	1,00					
Peso ao nascer (gramas)						
1.500-2.499	0,86	0,80-0,94	**0,001**			
2.500-3.999	1,00					
≥ 4.000	1,07	0,91-1,27	0,400			
Sexo da criança						
Masculino	1,05	0,99-1,12	0,128	1,06	0,99-1,13	0,098
Feminino	1,00					

*RP - Razão de Prevalência; IC95% - Intervalo de Confiança de 95%.*

Observa-se, na Tabela 2, que a variável peso ao nascer apresentou p<0,20, mas não foi incluída no modelo final, por apresentar colinearidade com a variável idade gestacional ao nascer. De maneira independente, nascer nas Regiões Norte (RP: 1,32; IC95%: 1,15-1,50; p: <0,001) ou Nordeste (RP: 1,23; IC95%: 1,09-1,39; p: 0,001), nascer em domicílio (RP: 1,30; IC95%: 1,04-1,63; p: 0,020), ser mãe de 4-7 anos de estudo (RP: 1,12; IC95%: 1,00-1,26; p: 0,049), gravidez múltipla (RP: 1,19; IC95%: 1,01-1,39; p: 0,036) e nascer de 28-31 semanas (RP: 1,31; IC95%: 1,19-1,49; p: <0,001) ou 32-36 semanas (RP: 1,08; IC95%: 1,00-1,16; p: 0,043) constituíram-se fatores de risco para o óbito evitável no período neonatal, comparando-se com crianças nascidas a termo. Foi fator de proteção para o óbito evitável nascer por cesariana (RP: 0,83; IC95%: 0,78-0,89; p: <0,001), comparado a nascer de parto vaginal.

Sobre a magnitude dos efeitos, no risco de óbito neonatal evitável, nascer nas Regiões Norte ou Nordeste aumentou de 32% e 23%, respectivamente, comparadas à Região Sul; 4-7 anos de escolaridade aumentou em 12%, comparado a 12 anos ou mais de escolaridade; sobre a ocorrência em domicílio, o risco foi 30% maior, quando comparada à ocorrência no hospital; sobre a gravidez múltipla, o risco foi 19% maior, quando comparada à gravidez única; e em relação a nascer prematuro, de 28-31 semanas ou 32-36 semanas, o risco foi de 33% e 8% maior, respectivamente, quando comparado a nascer a termo (Tabela 2).

A Tabela 3 refere-se às análises bruta e ajustada relativas ao óbito pós-neonatal evitável no ano de 2020.

**Tabela 3:** Regressão múltipla e regressão múltipla ajustada relativas aos óbitos pós-neonatais (n=1.422) evitáveis ocorridos em 2020 segundo dados do Sistema de Informação de Mortalidade, Brasil, 2020

Variável	Regressão múltipla			Regressão múltipla ajustada		
	RP	IC95%	Valor de *p*	RP	IC95%	Valor de *p*
Região						
Norte	0,78	0,63-0,97	0,022	0,78	0,63-0,96	0,021
Nordeste	0,92	0,77-1,11	0,392	0,92	0,77-1,11	0,383
Sudeste	0,98	0,83-1,15	0,782	0,97	0,82-1,15	0,737
Centro-Oeste	0,83	0,65-1,06	0,137	0,82	0,64-1,05	0,124
Sul	1,00					
Idade materna (anos)						
≤19	1,01	0,88-1,17	0,866	1,02	0,89-1,17	0,781
20-34	1,00					
35-40	0,88	0,75-1,03	0,111	0,87	0,74-1,02	0,095
>40	0,73	0,54-0,99	0,043	0,71	0,53-0,96	0,028
Cor/raça						
Indígena	1,32	0,97-1,81	0,079	1,35	0,99-1,85	0,058
Parda	1,14	1,01-1,29	0,032	1,14	1,01-1,28	0,040
Amarela	2,42	1,08-5,44	0,032	2,41	1,07-5,40	0,033
Preta	1,27	0,99-1,64	0,062	1,27	0,98-1,63	0,069
Branca	1,00					
Anos de escolaridade						
Nenhum	1,39	1,01-1,91	0,043	1,41	1,03-1,94	0,032
1-3	1,35	1,00-1,82	0,049	1,38	1,03-1,85	0,031
4-7	1,45	1,18-1,78	0,001	1,45	1,18-1,78	**<0,001**
8-11	1,30	1,08-1,57	0,006	1,30	1,08-1,57	0,005
≥ 12	1,00					
Local do óbito						
Outros	1,47	0,99-2,18	0,054	1,49	1,01-2,20	0,046
Via pública	1,69	1,03-2,79	0,039	1,68	1,02-2,77	0,042
Domicílio	1,57	1,33-1,86	**<0,001**	1,57	1,33-1,86	**<0,001**
Outros estabelecimentos	1,69	1,39-2,05	**<0,001**	1,69	1,40-2,05	**<0,001**
Hospital	1,00					
Número de filhos vivos						
≥ 4	0,97	0,79-1,20	0,809			
1-3	0,91	0,78-1,06	0,214			
0	1,00					
Tipo de gravidez						
Dupla ou tripla	1,30	1,01-1,68	0,042	1,28	0,99-1,64	0,056
Única	1,00					
Semanas de gestação						
22-27	1,12	0,42-3,03	0,819	1,04	0,39-2,79	0,937
28-31	1,60	1,19-2,16	0,002	1,42	1,08-1,88	0,013
32-36	1,11	0,96-1,27	0,161	1,02	0,90-1,16	0,716
37-41	1,00					
≥ 42	1,22	0,83-1,79	0,305	1,27	0,87-1,85	0,220
Tipo de parto						
Cesárea	0,84	0,76-0,94	0,003	0,84	0,75-0,94	0,002
Vaginal	1,00					
Peso ao nascer (gramas)						
1.500-2.499	0,86	0,75-0,98	0,029			
2.500-3.999	1,00					
≥ 4.000	1,14	0,87-1,49	0,332			
Sexo da criança						
Masculino	1,06	0,95-1,18	0,282			
Feminino	1,00					

* RP - Razão de Prevalência;

** IC95% - Intervalo de Confiança de 95%.

De acordo com a Tabela 3, a variável peso ao nascer apresentou p<0,20, mas não foi incluída no modelo final, por apresentar colinearidade com a variável idade gestacional ao nascer. No modelo final, de maneira independente, a criança ter raça/cor parda (RP: 1,14; IC95%: 1,01-1,28; p: 0,040) ou amarela (RP: 2,41; IC95%: 1,07-5,40; p: 0,033), a mãe não ter escolaridade (RP: 1,41; IC95%: 1,03-1,94; p: 0,032) ou ter de 1-3 anos (RP: 1,38; IC95%: 1,03-1,85; p: 0,031), 4-7 anos (RP: 1,45; IC95%: 1,18-1,78; p: <0,001) ou 8-11 anos (RP: 1,30; IC95%: 1,08-1,57; p: 0,005) de aprovação escolar, o óbito ter ocorrido em outros locais (RP: 1,49; IC95%: 1,01-2,20; p: 0,046), em via pública (RP: 1,68; IC95%: 1,02-2,77; p: 0,042), no domicílio (RP: 1,57; IC95%: 1,33-1,86; p: <0,001) ou outros estabelecimentos de saúde (RP: 1,69; IC95%: 1,40-2,05; p: <0,001) e a criança ter nascido com idade gestacional entre 28 e 31 semanas (RP: 1,42; IC95%: 1,08-1,88; p: 0,013) constituíram fator de risco para o óbito evitável no período pós-neonatal. Nascer na Região Norte (RP: 0,78; IC95%: 0,63-0,96; p: 0,021), idade materna acima de 40 anos (RP: 0,71; IC95%: 0,53-0,96; p: 0,028) e nascer de operação cesariana (RP: 0,84; IC95%: 0,75-0,94; p: 0,002) constituíram fator de proteção para o óbito pós-neonatal evitável.

Sobre a magnitude dos efeitos no óbito pós neonatal evitável, ter cor da pele parda aumentou em 14% e amarela, em 41% o risco; ser analfabeta, ter 1-3, 4-7 ou 8-11 anos de estudo aumentou o risco em 41%, 38%, 45% e 30%, respectivamente; ocorrência do óbito em outros serviços de saúde aumentou o risco em 69%, em via pública, em 68%, no domicílio, em 57% e em outros locais, em 49%, quando comparado ao óbito no hospital; nascer com idade gestacional entre 28-31 semanas resultou em risco 42% maior que nascer de termo. Sobre os fatores de proteção, crianças nascidas na Região Norte tiveram risco 22% menor em relação às nascidas na Região Sul; crianças cujas mães tinham 40 anos ou mais tiveram risco 29% menor, comparadas a mulheres na faixa etária entre 20-34 anos; e nascer de cesárea diminuiu o risco em 16%, comparando ao nascimento por parto vaginal (Tabela 3).

## DISCUSSÃO

O presente estudo permitiu identificar os fatores independentemente associados aos óbitos infantis evitáveis no ano de 2020, considerando seus componentes neonatal e pós-neonatal. Para os óbitos neonatais evitáveis, nascer de cesárea foi fator de proteção. Idade gestacional de 28-36 semanas, residir nas Regiões Norte ou Nordeste, escolaridade de 4-7 anos, óbito no domicílio e gravidez múltipla constituíram fatores de risco. Para o componente pós-neonatal evitável, nascer de cesárea foi fator de proteção, assim como nascer na Região Norte e idade materna acima de 40 anos. Os fatores de risco foram relacionados às escolaridades mais baixas, raça/cor amarela ou parda, óbito fora do hospital e nascer de 28-31 semanas de gestação.

Na presente investigação, residir nas Regiões Norte e Nordeste foi fator de risco para o óbito neonatal, concordando com outros estudos, os quais apontaram que as Regiões Norte e Nordeste concentram os piores índices de mortalidade neonatal do país^(19-20)^. Ainda, destaca-se que, no nordeste do Brasil, muitos estados apresentam comportamento estacionário em relação à mortalidade infantil, fato preocupante, visto que o índice de mortalidade ainda é alto^(21)^ e que, em Rondônia, na Região Norte, encontrou-se a redução mais relevante do país quando considerado o óbito neonatal evitável no período de 2000 a 2018^(22)^.

Nascer prematuro, de 28-36 semanas, foi fator de risco para o óbito neonatal evitável. Quando se aborda tal componente, a prematuridade está entre as causas dominantes, sendo considerada de difícil prevenção e dependente de diversos fatores, estando principalmente vinculada à qualidade do pré-natal, à organização dos serviços neonatais e ao preparo da equipe desde a Atenção Primária até a Atenção Terciária à Saúde, de forma que sua redução requer investimentos para o fortalecimento do sistema de saúde^(23)^. O acompanhamento pré-natal, quando realizado de forma adequada, é capaz de reduzir a morbimortalidade materno-infantil, havendo evidências de que o seguimento pré-natal completo e cuidados pós-natais estão relacionados à diminuição da mortalidade neonatal precoce^(24)^.

A prematuridade como causa de óbito infantil tem relevância no contexto mundial, pois, em 2022, contribuiu com 35% da mortalidade neonatal global^(25)^, sendo suas complicações as principais causas de morte em todas as regiões do mundo^(26)^, inclusive em países desenvolvidos. Na Sérvia, a taxa de mortalidade neonatal relacionada à prematuridade cresceu de 7,2% em 2000 para 11,9% em 2014^(27)^. Entre suas complicações, destacam-se a síndrome do desconforto respiratório e a asfixia ao nascer, visto que o desenvolvimento pulmonar fetal é interrompido na prematuridade^(28)^. Portanto, não é surpreendente que essas sejam as principais causas de morte neonatal no mundo, inclusive porque, além dos casos de prematuridade que ocorrem de forma natural, há também as condições médicas em que se faz necessário o abreviamento da gestação por questões maternas e/ou fetais. Dessa forma, apesar de complexa, visto seu caráter multifatorial^(29)^, é essencial a prevenção da prematuridade para que se obtenha redução dos óbitos neonatais evitáveis.

Ressalta-se, ainda, que o presente estudo utilizou uma classificação de evitabilidade do óbito infantil, que inclui apenas casos de crianças nascidas com pelo menos 1.500 g, o que certamente excluiu muitos casos de nascimentos prematuros, não aqui considerados passíveis de classificação segundo os critérios de evitabilidade adotados. Por outro lado, também merece destaque que a idade gestacional de 28-36 semanas constituiu fator de risco também para o óbito pós-neonatal, sendo que esse período, possivelmente, permitiu a inclusão de prematuros com peso igual ou superior a 1.500 g.

A gravidez múltipla foi associada ao óbito neonatal evitável, resultado concordante com o obtido em estudo coreano, sendo que ter gestação gemelar aumentou, em nove vezes, o risco de morte neonatal e, em seis vezes, o de morte infantil, de modo geral, quando comparado à gestação única, sendo que os autores apontaram que o risco aumenta exponencialmente em caso de trigêmeos ou quadrigêmeos^(30)^.

Ainda, merecem ser explorados em futuros estudos a escolaridade intermediária e 4-7 anos de aprovação escolar, por terem sido fatores de risco para o óbito neonatal evitável, resultado de difícil explicação, e óbito ocorrido no domicílio, pois, além da dificuldade de acesso em tempo hábil a serviços de saúde de qualidade^(31-32)^, outros aspectos precisam ser considerados no contexto da evitabilidade, como o tempo de gestação (prematuridade).

Globalmente, houve redução em 50% das mortes pós-neonatais gerais entre 1990 e 2015^(15)^. O óbito pós-neonatal tende a ter causas mais facilmente passíveis de intervenção, pois está relacionado às condições de vida e características familiares, como condição socioeconômica, educação, saneamento básico e água tratada, além do aumento de programas de saúde, cobertura vacinal e combate a doenças infecciosas transmissíveis^(33-34)^. Nesse sentido, os fatores associados encontrados neste estudo que guardam relação com aspectos sociais são escolaridade e óbito fora do hospital, pela relação com acesso aos serviços de saúde.

Ter cor da pele/raça parda ou amarela constituiu fator de risco para o óbito pós-neonatal, sendo que, na vigência da cor amarela, houve duas vezes mais risco de morte, fato que precisa ser visto com cautela, pelo pequeno número de casos (n=7) e porque estudos trazem menor taxa de mortalidade nessa população^(35-36)^. Nos Estados Unidos da América, crianças pretas morrem três vezes mais do que crianças brancas^(37)^, relação não observada no presente estudo. Em estudo brasileiro, realizado no estado de Pernambuco, observou-se que crianças pretas e pardas estavam 13 vezes mais associadas ao óbito neonatal^(38)^ e, no Mato Grosso do Sul, crianças pardas apresentaram aumento dos óbitos neonatais entre 2005 e 2013^(39)^.

Encontrou-se que nascer de cesárea foi fator de proteção para o óbito evitável, tanto quando considerado o componente neonatal quanto o pós-neonatal, sendo esse resultado concordante com o obtido por estudo que teve como foco a comparação entre as vias de parto e encontrou que, em geral, a maioria das mortes neonatais ocorreu após o parto vaginal, podendo estar associada a práticas assistenciais^(40)^. É possível que o uso de procedimentos e intervenções danosas durante o parto possa aumentar o risco de morte da criança, mas também as cesarianas sem indicação podem interromper a gestação previamente, consequentemente aumentando o número de nascidos prematuros^(41)^. Estudos identificaram o mesmo fator protetor da cesariana na mortalidade infantil em geral, ou seja, sem considerar os critérios de evitabilidade^(42-43)^. No entanto, não foi possível, neste estudo, identificar se a cesárea foi eletiva ou de emergência, condição essencial para que se compreenda exatamente o papel protetor da cesárea.

Para o componente pós-neonatal, residir na Região Norte protegeu do óbito evitável, situação que merece ser mais bem investigada em futuros estudos. A idade materna superior a 40 anos também protegeu da ocorrência de óbito evitável, ou seja, os óbitos que ocorreram nessa faixa etária, em sua maioria, não foram por causas evitáveis. Isso pode ocorrer devido à idade materna avançada estar associada a maior risco de anormalidades congênitas e cromossômicas, que resultam em complicações fetais^(44)^. Destaca-se que, na maioria dos casos, a associação entre malformação e morte infantil é de difícil prevenção, sendo controversa a indicação de triagem e interrupção precoce, que é, muitas vezes, a única opção^(45)^. Diferentemente do presente estudo, em estudo coreano, observou-se que a idade materna superior a 40 anos estava associada a maior risco de morte infantil, comparação que precisa ser feita com cuidado, visto que, no caso da Coreia, abordou-se o óbito em geral e não segundo critérios de evitabilidade^(46)^.

### Limitações do estudo

Destaca-se como fragilidade deste estudo o fato de ter utilizado banco de dados secundário, o que não permite o controle da coleta de dados. Porém, apesar da necessidade de exclusão de parte dos casos, a base efetivamente utilizada ainda se manteve grande, indicando, possivelmente, um estimador não viciado. Considera-se potência a ser destacada o fato de ter sido usado um banco de dados de base populacional de um país como o Brasil, que tem dimensões continentais. Outra fragilidade a ser apontada é o fato de ter-se trabalhado com a proporção de óbitos evitáveis, não sendo calculadas as taxas de mortalidade, que são um indicador frequentemente utilizado nos estudos sobre essa temática.

### Contribuições para a área de enfermagem

Estudos sobre a evitabilidade do óbito são relevantes para a área da enfermagem, visto que o enfermeiro tem papel essencial na investigação do óbito infantil e na classificação da evitabilidade do óbito nos comitês de mortalidade. Assim, os resultados obtidos sinalizam a potência da área de enfermagem na proposição e execução de políticas públicas em saúde que englobam acesso adequado e de qualidade, desde a gestação até a primeira infância, ampliando o olhar para essa população e articulando com as tecnologias disponíveis no sistema de saúde, com o intuito de contribuir para a redução da mortalidade infantil evitável no Brasil.

## CONCLUSÕES

Os resultados permitiram identificar os determinantes que se associaram aos óbitos neonatais e pós-neonatais. Para os óbitos neonatais evitáveis, nascer de cesárea foi fator independente de proteção, enquanto que a prematuridade constituiu fator de risco. Residir nas Regiões Norte e Nordeste, ter baixa escolaridade e a ocorrência de óbito em domicílio também constituíram fatores de risco independentes para o desfecho negativo. No período pós-neonatal, escolaridade materna, óbitos ocorridos fora do hospital e raça/cor parda e amarela constituíram fatores de risco para óbito evitável. Nascer por cesárea foi fator protetivo independente, assim como residir na Região Norte e idade materna acima de 40 anos.

Considerando a magnitude dos efeitos, destacou-se, para o óbito neonatal evitável, a prematuridade. Para o óbito pós-neonatal evitável, destacou-se o risco social, representado pela baixa escolaridade materna e dificuldade de acesso aos serviços de saúde, evidenciado pela ocorrência do óbito fora do ambiente hospitalar.
